# Scientometric evaluation of highly cited scientists in the field of forensic science and legal medicine

**DOI:** 10.1007/s00414-020-02491-x

**Published:** 2021-01-02

**Authors:** Alan Wayne Jones

**Affiliations:** Division of Drug Research, Department of Biomedical and Clinical Sciences, Linköping University, Linköping, Sweden

**Keywords:** Bibliometrics, Citation analysis, Forensic science, Legal medicine, Publications

## Abstract

A publically available database of the most highly cited scientists in all disciplines was used to identify people that belonged to the subject category “forensic science and legal medicine.” This bibliometric information was derived from Elsevier’s SCOPUS database containing eight million scientists with at least five articles as author or co-author. The top 100,000 most highly cited scientists were identified and ranked according to six citation metrics; total number of citations, H-index, H-index adjusted for co-authorship, citations to single-authored papers, citations to single or first author papers and, citations to single, first, or last-authored papers. The eight million entries in the SCOPUS database were sub-divided into 22 main subject categories and 176 sub-categories, one of which was legal and forensic medicine. The citation databases were provided as supplementary material in two articles published in PLoS Biology in 2019 and 2020. Among the top 100,000 most highly cited scientists, there were only 30 allocated to the legal and forensic medicine category, according to the 2019 PLoS Biology article. The updated database from 2020 also included the names of people within the top-cited 2% of their scientific discipline. This increased the number of forensic practitioners to 215 from a total of 10,158 individuals in this subject category. This article takes a closer look at these highly cited forensic scientists, the countries where they work, the particular research field in which they publish, and their composite citation scores with and without self-citations. The top ten most cited individuals in both databases (2019 and 2020) were the same and these should therefore be considered an elite group among all forensic practitioners.

**Supplementary Information:**

The online version contains supplementary material available at 10.1007/s00414-020-02491-x.

## Introduction

Forensic science is a multidisciplinary subject, which encompasses many different branches of science, and medicine, as exemplified by the 11 sections of the American Academy of Forensic Sciences (AAFS) with a current membership of over 6500: anthropology, criminalistics, digital and multimedia sciences, engineering and applied sciences, general, jurisprudence, odontology, pathology/biology, psychiatry and behavioral sciences, questioned documents, and toxicology. Forensic practitioners submit the results of their research for publication to a wide range of scientific journals, not only forensic journals. However, most practitioners tend to publish articles in multidisciplinary journals, such as Journal of Forensic Sciences (Wiley-Blackwell), the first volume of which appeared in 1956, or Forensic Science International (Elsevier) which began publishing in 1972.

Also available are many more specialized forensic journals that focus on various sub-disciplines, such as Journal of Analytical Toxicology (Oxford), Forensic Toxicology (Springer), International Journal of Legal Medicine (Springer), and Academic Forensic Pathology (SAGE). Accordingly, there is no shortage of journals from well-established publishers where forensic practitioners can submit their work for peer review and publication and in this way spread new knowledge and ensure that this information enters the public domain.

Accumulating a long list of publications in high-impact scientific journals is considered meritorious in academia and scholarly publishing [1, 2]. This is particularly beneficial in university, such as when people apply for promotion or tenure and when research funding decisions are made. A strong publication track record is important and is always one of the key consideration when people apply for research grants and scholarships [3–5]. However, the raw number of publications can be misleading when judging the merits of a person’s work, without also considering the pattern of authorship and impact factors of the journals [6]. The total number of articles listed in a person’s CV gives an indication of productivity, but quantity is not the same as quality and some people fail to differentiate between conference abstracts, posters, letters-to-the-editor, case reports, editorial material, original research articles, reviews, and book chapters, all of which are combined together in the same publication list.

Another dilemma is the rise in multi-authored papers, which makes it difficult to know exactly what each person listed as an author actually contributed to completion of the work [7, 8]. However, there is broad agreement that the first name on a multi-authored paper is more significant and this person deserves extra credit compared with other names listed among the co-authors. In collaborative research, the last name on a paper is usually the team leader or the professor of the department where the research was done [9]. What other names on a paper have contributed is an open question, although most journals now require a declaration about the role played by the various co-authors.

The quality and usefulness of a published article can only be properly assessed by carefully reading its contents to determine what relevance it might have in the planning and execution of your own future research endeavors. However, a widely accepted surrogate measure of a paper’s usefulness is the number of times the work is subsequently cited in articles penned by other scientists [10]. Counting and evaluating citations is an important part of library and information science, which is sometimes referred to as “the currency of science.” This entails documentation of scholarly publications, in terms of authorship of the articles, the journals where the work was published and the number of citations received [11].

In this article, I used two citation databases developed by scientists from Stanford University listing the most cited scientists in all subject disciplines. This information was contained in supplementary EXCEL files linked to the two articles published in PLoS Biology [12, 13]. A novel feature of the databases was that citations to single-author, first- or single-author, and first-, single-, or last-author papers were considered when a composite citation score was calculated. I searched and filtered this information to find the names of highly cited authors in the field of forensic science and legal medicine.

## Citation databases

The PLoS Biology article published in 2019 was entitled “A standardized citation metrics author database annotated for scientific field” with John PA Ioannidis as the lead author [12]. This research group from Stanford University used Elsevier’s SCOPUS database to extract information about the publications and citations to eight million scientists each of whom had authored or co-authored at least five entries in the database. The eight million scientists and their publications represented 22 large scientific fields (e.g., chemistry, biology, clinical medicine) and 176 sub-fields (e.g., immunology, substance abuse, legal and forensic medicine). The 2019 article included citation data covering a 22-year period from January 1996 to December 2017. For papers published between 1960 and 1995, the citations received in 1996–2017 were included in the calculations, but not the citations received by these articles up to 1995.

From this massive database of information, the 100,000 most highly cited scientists representing all scientific disciplines were selected and rank-ordered after a composite score. The latter was derived using six different citation metrics: (i) total number of citations, (ii) the person’s H-index [14], (iii) H-index adjusted for co-authorship [15], (iv) citations to papers as a single author, (v) citations to single- or first-author papers, and (vi) citations to single-, first-, or last-author papers. The composite scores were then calculated with and without including self-citations. A self-citation occurs when a person cites a paper on which he or she was listed as a co-author. In some fields, self-citation rates are higher than others [16]. The H-index is the number of articles in a person’s bibliography that have received at last H citations [17] and an adjusted H-index taking into consideration the number of co-authors [15].

In October 2020, the Stanford University group published a new article in PLoS Biology entitled “Updated science-wide author database of standardized citation indicators” which contained citation date up to the end of 2019 [13]. A new feature of this latest version was the inclusion of the names of scientists if they were within the top-cited 2% of all people publishing in their particular subject category. The updated database included citation data for over 160,000 scientists and the number of scientists that publish articles with that particular scientific discipline, such as legal and forensic medicine.

## Highly cited forensic scientists

 One of the EXCEL files provided as supplementary material was labeled S-1, and contained citation date up to end of 2017. Another supplementary file S-4 contained similar information up to end of 2018, but I noted some discrepancies in the publications count so the present breakdown of the data is based on the EXCEL file S-1. Overall, there was a remarkably good agreement between the names of people in both lists (S-1 and S-4). The information was sorted and filtered in various ways and there were 30 forensic practitioners among the top 100,000 most highly cited scientists in all scientific disciplines.

The names of these 30 people are listed in Table 1 and they are rank ordered after their position among the top 100,000 scientists in all subject categories. Also shown in the table is the country where they work, their special area of forensic expertise, number of papers in the SCOPUS database, and their composite citation scores with and without including self-citations.

**Table 1:** Names of the 30 forensic scientists ranked among the top 100,000 highly cited scientists in all subject categories, their country, specialty area, number of papers, and their composite citation scores calculated with and without including self-citations. Information derived from the EXCEL file S-1 from the PLoS Biology article [12]

Scientist	Country	Specialty area^1^	Paper count	Rank^2^	Composite score (NSC)^3^	Composite score (WSC)^4^
Kintz, P.	France	Toxicology	455	9321	4.1696	4.2111
Jones, AW.	Sweden	Toxicology	277	17,907	3.9869	4.0647
Drummer, OH.	Australia	Toxicology	263	18,033	3.9847	4.0084
Byard, RW	Australia	Pathology	796	22,663	3.9173	4.1776
Budowle, B.	USA	Genetics/DNA	497	23,807	3.9027	3.9769
Brinkmann, B.	Germany	Pathology/genetics	460	24,376	3.8952	3.9447
Kayser, M.	Netherlands	Genetics/DNA	235	24,642	3.8920	3.9839
Butler, JM.	USA	Genetics/DNA	137	26,368	3.8710	3.9255
Gill, P.	Norway	Genetics/DNA	141	27,761	3.8542	3.9270
Madea, B.	Germany	Pathology	695	33,460	3.7945	3.9025
Mitchell, RJ.	Australia	Genetics/DNA	386	38,688	3.7465	3.8170
Stuart, BH.	Australia	Taphonomy	96	49,777	3.6602	3.7155
Musshoff, F.	Germany	Toxicology	225	50,015	3.6586	3.6915
Pounder, DJ.	UK	Pathology	167	53,898	3.6319	3.6328
Işcan, MY.	Turkey	Anthropology	63	60,551	3.5893	3.6097
Ubelaker, DH.	USA	Anthropology	121	63,069	3.5743	3.6096
Pragst, F.	Germany	Toxicology	123	63,826	3.5701	3.6032
Logan, BK.	USA	Toxicology	106	67,721	3.5475	3.5632
Evett, IW.	UK	Statistics	111	70,868	3.5299	3.5747
Milroy, CM.	Canada	Pathology	82	73,861	3.5143	3.5318
Skopp, G.	Germany	Toxicology	170	75,982	3.5036	3.5489
Roewer, L.	Germany	Genetics/DNA	123	82,690	3.4703	3.5737
Parson, W.	Austria	Genetics/DNA	303	83,131	3.4682	3.6430
Püschel, K.	Germany	Pathology	748	85,739	3.4565	3.5205
Karch, SB.	USA	Pathology	82	90,686	3.4342	3.4582
Tsokos, M.	Germany	Pathology	339	93,819	3.4201	3.5363
Carracedo, A.	Spain	Genetics/DNA	691	101,180	3.3893	3.4910
Morling, N.	Denmark	Genetics/DNA	407	102,756	3.3830	3.4928
Thali, MJ.	Switzerland	Pathology/imaging	349	107,335	3.3650	3.5140
Pollak, S.	Germany	Pathology	264	117,197	3.3278	3.4946

^1^Based on a search of PUBMED and review of actual published articles

^2^Rank among the top 100,000 most cited scientists in all scientific disciplines

^3^*NSC* no self-citations counted

^4^*WSC* with self-citations counted

Looking at Table 1, one notices that 14 countries were represented: Germany (*n* = 9), USA (*n* = 5), Australia (*n* = 4), the UK (*n* = 2), and one person from each of the ten other countries listed. The average number of papers published by these 30 scientists according to SCOPUS was 297 with a range from 63 to 796. The areas of forensic science represented were dominated by genetics/DNA sequencing, forensic pathology, and analytical and forensic toxicology. The overall ranking shows that only 12 of the 30 names in Table 1 reached the top 50,000 among all 100,000 scientists representing all disciplines and one person reached the top 10,000 (rank 9321).

## Expanded list of highly cited scientists

The 2020 PLoS Biology article updated the previous citation database to include citations up to the end of 2019. In addition, it included the names of scientists if they were among the top-cited 2% within their scientific discipline. The number of forensic practitioners represented now increased from 30 to 215 and the total number of scientists within this discipline was 10,158. The average number of papers produced by these 215 scientists was 145 with a range from 16 to 887 papers. However, simply looking at the number of publications is not very informative because people began publishing, depending on their age, at different time periods.

Table 2 gives a breakdown of the 215 top-cited forensic scientists according to the country where they worked, according to information on recent published articles. Obviously, people sometimes change their jobs and countries, so this must be considered when evaluating the distribution of countries where the highly cited work was actually done. There were *n* = 31 countries represented and topping the list was the USA with *n* = 46 highly cited scientists followed by Germany (*n* = 32), Great Britain (*n* = 27), Australia (*n* = 19), Canada (*n* = 11), and Japan (*n* = 10). The data in the table also normalizes for the population in these countries, and Norway and Switzerland then ranked highest, owing to their low population density.

**Table 2:** Distribution of 215 highly cited forensic scientists according to the countries where they are based. These individuals were within the top-cited 2% of all scientists in that scientific discipline (*n* = 10.158 practitioners)

Country	*n*^1^	Population in millions	Number per million inhabitants
USA	46	331.0	0.139
Germany	32	83.7	0.382
Great Britain	27	67.9	0.398
Australia	19	24.2	0.785
Canada	11	37.7	0.292
Japan	10	126.5	0.079
Switzerland	8	8.7	0.92
Sweden	7	10.1	0.693
Norway	6	5.4	1.11
France	6	65.3	0.092
Finland	4	5.5	0.727
Poland	4	37.9	0.106
Spain	4	46.7	0.086
Italy	4	60.5	0.066
Netherlands	4	17.1	0.234
India	3	1380.0	0.002
Denmark	3	5.8	0.517
Austria	2	9.0	0.222
Portugal	2	10.2	0.196
New Zealand	2	4.8	0.417
South Africa	2	59.3	0.034
Israel	1	8.7	0.115
Iran	1	84.0	0.012
Mexico	1	129.9	0.008
Czech Republic	1	10.7	0.093
Brazil	1	212.6	0.005
Ireland	1	4.8	0.208
China	1	1439.3	0.0007
Turkey	1	84.3	0.012
Singapore	1	5.9	0.169
Qatar	1	2.9	0.345

^1^Number of scientists listed in the database

^2^Country population size according to a GOOGLE search

## Forensic science elite

As expected, the forensic scientists listed in Table 1 were also among the expanded highly cited list of 215 names in the updated database. The names of the top ten individuals from this expanded dataset are shown in Table 3 along with their country and the university/institute where they were based. Also shown is the number of publications they were credited with in SCOPUS, year of first and last publication, rank order within the entire population of eight million publishing scientists, and their composite scores calculated with and without including self-citations.

**Table 3:** The top ten most highly cited scientists in forensic science and legal medicine among the most highly cited scientists in all scientific disciplines derived from the PLoS Biology article reference [13]

Scientist	Institute/university	Country	Paper count	Publication years	Rank^1^	Composite score^2^ (with self-cites)
Kintz, P.	University of Strasbourg	France	500	1988–2020	10,321	4.14 (4.20)
Gill, P.	University of Oslo	Norway	188	1989–2020	16,824	4.01 (4.07)
Kayser, M.	Erasmus MC, Netherlands	Netherlands	283	1995–2020	19,499	3.97 (4.06)
Drummer, OH.	Monash University	Australia	285	1976–2020	19,623	3.97 (3.99)
Jones, AW.	University of Linköping	Sweden	294	1974–2019	20,065	3.96 (4.04)
Byard, RW.	University of Adelaide	Australia	887	1985–2020	20,467	3.95 (4.19)
Butler, JM.	National Institute of Standards and Technology	USA	144	1994–2020	22,795	3.92 (3.97)
Budowle, B.	University of North Texas Health Sciences Center	USA	566	1981–2020	24,019	3.91 (3.98)
Brinkmann, B.	Institute of Forensic Genetics	Germany	455	1969–2015	27,591	3.87 (3.91)
Madea, B.	University of Bonn	Germany	730	1984–2020	27,949	3.86 (3.95)

^1^Rank among the top 100,000 most highly cited scientists in all disciplines

^2^Composite score was derived from six citation metrics: (i) total citations, (ii) H-index, (iii) H-index adjusted for co-authorship, (iv) citations to single-author papers, (v) citations to single- or first-author papers, (vi) citations to single-, first-, or last-author papers

Table 4 shows the six citation metrics for this elite group of highly cited forensic practitioners that were used to calculate a composite score. The numbers in brackets are the number of single-, first-, or last-author papers listed in SCOPUS. Each of the six citation metrics was weighted in relation to a maximum score for each metric and added together to give the composite citation score. For more details of the algorithm used in this calculation, I refer to the original articles in PLoS Biology [12, 13].

**Table 4:** Summary of the six citation metrics used to calculate the composite scores shown in Table 3 for the top-ten most highly cited scientists in the discipline forensic science and legal medicine without counting self-citations

Scientist	Paper count	Total citations	Hirsch index	Adjusted H-index	Single author cites (*n*)^1^	Single or first author cites (*n*)^1^	Single, first or last author cites (*n*)^1^
Kintz, P,	500	8834	49	33.19	983 (64)	4527 (259)	6197 (340)
Gill, P.	188	9890	57	22.11	470 (12)	3170 (61)	6846 (129)
Kayser, M.	283	14225	65	18.87	247 (13)	2652 (45)	7374 (153)
Drummer, OH.	285	7350	45	26.96	794 (37)	2070 (88)	4805 (199)
Jones, AW.	294	4460	36	28.23	1315 (108)	3146 (218)	4041 (265)
Byard, RW.	887	7892	35	22.82	780 (202)	3539 (497)	5915 (809)
Butler, JM.	144	6317	47	21.86	922 (21)	2270 (55)	3893 (95)
Budowle, B.	566	11841	55	24.44	85 (22)	3380 (130)	7312 (347)
Brinkmann, B.	455	8485	44	24.77	335 (19)	1574 (104)	5050 (344)
Madea, B.	730	7279	43	26.91	352 (55)	1229 (178)	5772 (591)

^1^*n*, number of papers as single, first, and last author

^2^Calculated based on six citation metrics: (i) total citations, (ii) Hirsch or H-index, (iii) H-index adjusted for co-authorship, (iv) citations to single author papers, (v) citations to single or first author papers, (vi) citations to single, first or last author papers

## Discussion

The present article is not the first to investigate citation records of forensic scientists, because a paper from 2005 was entitled *Crème de la Crème in forensic science and legal medicine: the most cited articles, authors and journals 1981–2003* [18]. This earlier article was based on information available from the Web-of-Science database (Thomson Reuters, Philadelphia) which only considered total number of citations to published papers by that author and no consideration was given to whether a person was single, first, or last author. Interestingly, several of the people identified in the 2005 article are also included in the present compilation of highly cited scientists, namely Budowle, Brinkmann, Gill, Kintz, Carracedo, and Roewer. This testifies to their sustained contribution and that they continue to publish important papers that were highly cited during the past 15 years since 2005.

The present article focused on highly cited scientists in some branch of the forensic sciences and legal medicine as their primary area of research. There were only 30 such individuals (Table 1) among the 100,000 most highly cited scientists in all scientific disciplines. When the selection criteria were expanded to include people within the top-cited 2% of forensic practitioners (2020 article in PLoS Biology), the number of forensic practitioners increased to 215 from a total of 10,158 people considered to belong to the discipline of legal and forensic medicine.

A novel feature of the PLoS Biology articles was an attempt to balance citations with the relative position of an author’s name on the published article, whether single author, first author, or last author. Other citation databases, such as Web-of-Science, credit every author on a paper with all the citations that it might receive. If a few multi-author collaborative studies accrue masses of citations, this can skew the citation scores for some of the names on the article, who might only publish a few paper during their entire career. With single-author papers, which are rapidly diminishing in most science journals, it is obvious to whom the credit and responsibility belongs [19]. The phenomenon of honorary and ghost authorship is also a growing problem in academic publishing that cannot be ignored [20].

Some might not agree with this strategy, but there is wide consensus that the first name on a multi-authored paper deserves more credit than the other names on the paper [21]. The last name listed on a paper is often the senior author, research group leader, or sometimes the head of the university department where the work was done. The person listed as corresponding author, usually the first or last name on the article, also deserves special mention, because they receive comments and critique from the peer reviewers and re-submit a revised manuscript. The H-index, which is a widely used metric to compare and contrast the work of different scientists, has been updated to account for multiple authorship [15].

An evaluation of highly cited articles, authors, and institutions specializing in “forensics” was the subject of an article published in a newsletter called ScienceWatch produced by Thomson Reuters (Philadelphia, USA). Their survey covered the years 2001 to 2011 and was based on citation data from Web-of-Science, which only considers the total number of citations to published articles. Accordingly, a multi-national collaborative study, e.g., concerning forensic genetics or DNA that might have scores of names on the published article, can inflate the citation record for individual scientists. The ScienceWatch article identified John Butler (NIST) as the person with highest citation impact and he is also included in the current listing based on six citation metrics so he is evidently still producing important articles that are highly cited.

The SCOPUS database contains citation data covering a 23-year period from January 1996 to December 2019, which is representative of most living scientists, who are still active and publish their work in mainstream scientific journals. There was a strong correlation between the information in the two databases from 2017 (S-1) and the updated version in the 2020 publication [13]. When comparing the two databases, the same people were included in both compilations, although there was a slight change in their relative rank ordering.

It is important to note that only articles indexed by SCOPUS were considered when the citation metrics were derived, so authors of books or book chapters that might have been highly cited are not credited for this work. Forensic science and legal medicine are closely linked with law enforcement and criminal investigations, so it would be of interest to know how often a person’s published articles are cited or used in actual court cases that involve presentation and interpretation of scientific evidence [22]. But such information is a lot more difficult to document.

The citation databases used to prepare the present article are in the public domain and were made available as supplementary material to the two PLoS Biology articles [12, 13] and these are also posted on the open data repository Mendeley (https://data.mendeley.com). The names and citation metrics for the 215 most highly cited researchers publishing in the domain of “legal and forensic medicine” are contained in supplementary EXCEL file Supp-1. According to the PLoS Biology article, this was their primary research discipline. However, there were 76 people with “legal and forensic medicine” as their secondary subject discipline and their names and citation scores are found in supplementary EXCEL file Supp-2.

## Supplementary information

ESM 1 (XLSX 93.2 kb)

ESM 2 (XLSX 39 kb)
